# Evaluating Antimicrobial Resistance Trends in Commensal *Escherichia coli* Isolated from Cecal Samples of Swine at Slaughter in the United States, 2013–2019

**DOI:** 10.3390/microorganisms11041033

**Published:** 2023-04-15

**Authors:** Hamid Reza Sodagari, Csaba Varga

**Affiliations:** 1 Department of Pathobiology, College of Veterinary Medicine, University of Illinois Urbana-Champaign, Urbana, IL 61802, USA; 2 Carl R. Woese Institute for Genomic Biology, University of Illinois Urbana-Champaign, Urbana, IL 61802, USA

**Keywords:** antimicrobial resistance, multidrug resistance, pigs, surveillance, cecal samples, slaughter, United States

## Abstract

The emergence of antimicrobial resistance (AMR) in commensal and pathogenic enteric bacteria of swine is a public health threat. This study evaluated publicly available AMR surveillance data collected by the National Antimicrobial Resistance Monitoring System (NARMS) by assessing AMR patterns and temporal trends in commensal *E. coli* isolated from cecal samples of swine at slaughter across the United States. We applied the Mann-Kendall test (MKT) and a linear regression trend line to detect significant trends in the proportion of resistant isolates to individual antimicrobials over the study period. A Poisson regression model assessed differences among years in the number of antimicrobials to which an *E. coli* isolate was resistant. Among the 3237 *E. coli* isolates, a very high prevalence of resistance for tetracycline (67.62%), and high resistance for streptomycin (24.13%), and ampicillin (21.10%) were identified. The MKT and the linear trend line showed a significantly increasing temporal trend for amoxicillin-clavulanic acid, ampicillin, azithromycin, cefoxitin, ceftriaxone, and trimethoprim-sulfamethoxazole. Compared to 2013 the number of antimicrobials to which an *E. coli* isolate was resistant was significantly higher in the years 2017, 2018, and 2019. The increasing temporal trend of resistance to important antimicrobials for human medicine (e.g., third-generation cephalosporins) and the increase in multidrug resistance in the later years of the study are concerning and should be followed up by studies to identify sources and risk factors for the selection of AMR.

## 1. Introduction

The emergence of antimicrobial resistance (AMR) in commensal and pathogenic bacteria has become a global health concern that requires a One Health approach as human, animal, and environmental health are interconnected [1]. Moreover, infections with antimicrobial-resistant bacteria increase therapeutic failures and costs and can also be spread from livestock to humans [2,3].

In the United States of America (US) the Centers for Disease Control and Prevention (CDC) highlighted the importance of combating AMR as more than 2.8 million antibiotic-resistant infections occur each year, and more than 35,000 people die as a result [4]. To combat the emergence of AMR in animal populations, in 2018, the U.S. Food and Drug Administration (FDA), Center of Veterinary Medicine defined main priorities, including supporting antimicrobial stewardship programs by advocating for judicious antimicrobial use in veterinary settings and improving antimicrobial use (AMU) and AMR monitoring in animals [5].

The widespread use of antimicrobials in livestock, including on swine farms, is one of the main drivers for the emergence of AMR [6,7]. According to the FDA estimate, livestock production accounted for almost 70% of all medically important antibiotic sales in the United States in 2021, and the highest proportion (42%) of these antimicrobials has been used in swine [8]. In response to national and global mandates, the livestock sector in the US is proactively implementing AMU reduction strategies and progressively eliminating the preventive use of medically important antimicrobials to contain the emergence of AMR. In 2012, FDA published its first Guidance for Industry (GFI) #209 [9] which stipulated limiting the use of medically important antimicrobials only for disease prevention, treatment, and control, and the use of these antimicrobials should be under veterinarian oversight. In addition, the use of antimicrobials as growth promoters was not allowed. An additional GFI #213 was fully implemented in January 2017 that required veterinary oversight of medically important antimicrobials administered via feed or water, and each administration requires a Veterinary Feed Directive (VFD) order (for in-feed use) or prescription (for antimicrobials administered via water) [10].

Swine meat and products are one of the most important sources of protein worldwide [11]. The US was the world’s third-largest producer and consumer of swine and swine products after the European Union and China in 2022 [7,12,13]. This high level of consumption further underscores the important potential role of swine meat and products in the dissemination of resistant commensal and/or pathogenic bacteria through human food consumption in the United States.

Since swine meat and products might be potential sources of human exposure to resistant commensal and pathogenic bacteria [14] monitoring the trends of AMR at the farm [7,13] and slaughter [15,16] levels is necessary to identify emerging trends to prevent human infections caused by foodborne antimicrobial-resistant bacteria. 

Commensal *Escherichia coli* (*E. coli*) are ubiquitous bacteria in the intestinal tract of humans and warm-blooded animals [17], and are good indicators of fecal contamination of meat [18], as they can contaminate meat and meat products during slaughter (i.e., evisceration), after slaughter, during the handling of meat, or through tainted water [11,19]. *Escherichia coli* are also a good indicator of the selective pressure imposed by antimicrobial use in livestock [20], as they can acquire and transfer resistant genes to other commensal and pathogenic bacteria in the intestinal tract and may pose a risk to human and animal health [21,22]. Commensal *E. coli* are commonly used in AMR monitoring programs of livestock, humans, and their environment as they are easy and economically feasible to isolate [23,24].

Surveillance systems are vital to monitor the AMR status and change over time of commensal and pathogenic bacteria to identify emerging trends to support public health authorities in implementing effective mitigation measures [25,26]. Good examples of surveillance programs that include monitoring of AMR in indicator bacteria from swine are the National Antimicrobial Resistance Monitoring System (NARMS) in the USA, the Danish Integrated Antimicrobial Resistance Monitoring and Research Program (DANMAP) in Denmark, the European Antimicrobial Resistance Surveillance Network in Veterinary Medicine (EARS-Vet) in Europe, and the Canadian Integrated Program for Antimicrobial Resistance Surveillance (CIPARS) in Canada [27,28,29,30].

Long-term monitoring of AMR trends in commensal bacteria from swine at slaughter is useful to assess the effectiveness of antimicrobial stewardship programs and to measure the potential human health risk posed by antimicrobial-resistant bacteria [31]. To address this, the present study evaluated NARMS data to determine the prevalence and phenotypic AMR patterns and trends of commensal *E. coli* isolated from the cecal content of market swine and sows at slaughter in the US between 2013 and 2019.

## 2. Materials and Methods

### 2.1. Study Design

The present study used the publicly available food animal surveillance data of cecal samples collected from market swine and sows at slaughter facilities throughout the US between 2013 and 2019 by the United States Department of Agriculture (USDA) Food Safety Inspection Service (FSIS) under the National Antimicrobial Resistance Monitoring System (NARMS) Surveillance Program using published methods [28]. In brief, to obtain a representative sample of overall national swine production, cecal samples from individual market hogs and sows are collected randomly considering the establishment size, animal classes slaughtered, and annual slaughter volumes.

### 2.2. Bacterial Isolation and Antimicrobial Susceptibility Testing

Standard microbiological techniques were applied to isolate *E. coli* from cecal swine samples at the USDA FSIS Eastern Laboratory [32].

The NARMS interpretive criteria for susceptibility testing and classification of resistant isolates were based on the minimum inhibitory concentrations (MIC) breakpoints established by CLSI (Clinical and Laboratory Standards Institute) M100-Ed30 document, and the CLSI MIC Interpretive Standards (M100-S29), except for streptomycin, and azithromycin, which has no CLSI breakpoint. The following antimicrobials and their breakpoints were included: amoxicillin-clavulanic acid (AMC) (≥32/16 μg/mL), ampicillin (AMP) (≥32), azithromycin (AZM) (≥32), cefoxitin (FOX) (≥32), ceftriaxone (AXO) (≥4), chloramphenicol (CHL) (≥32), ciprofloxacin (CIP) (≥0.12), gentamicin (GEN) (≥16), nalidixic Acid (NAL) (≥32), streptomycin (STR) (≥64 before 2014; ≥32 after 2014), tetracycline (TET) (≥16), and trimethoprim-sulfamethoxazole (COT) (≥4/76).

Intermediate isolates were classified as susceptible. Resistance to antimicrobials was defined as rare: <0.1%, very low: 0.1% to 1.0%, low: >1.0% to 10.0%, moderate: >10.0% to 20.0%, high: >20.0% to 50.0%, very high: >50.0% to 70.0% and extremely high: >70.0% [33].

### 2.3. Statistical Analyses

Statistical analyses were performed using STATA Intercooled software (Version 17, Stata Corporation, College Station, TX, USA) and R software [34] in the RStudio platform (Version 1.4.1106 © 2009–2021 RStudio, PBC). For each antimicrobial, the proportion of resistant isolates was calculated by dividing the number of isolates resistant by the total number of isolates tested.

The Mann–Kendall test (MKT) and a slope constructed by regressing the mean resistance over the study period (2013–2019) were used to identify monotonous temporal trends of AMR. The slope indicates the increasing or decreasing trend and its gradient, while the MKT identifies the significance of trends in a time series.

A single-linkage clustering dendrogram was constructed using Ward’s hierarchical clustering method with Euclidean distances to assess co-resistance patterns of isolates and was illustrated in a heatmap using the heatmap.2 package with ggplots and RColorBrewer libraries in R software. To assess correlations between antimicrobials Pearson correlation coefficients were calculated and illustrated in a correlogram (correlation matrix).

A Poisson regression model was constructed for the count outcome variable signified by the number of antimicrobials to which an *E. coli* isolate was resistant. The predictor variable was signified by the year of sampling, where 2013 was used as a referent to which all the other years (2014–2019) were compared. For the regression model incidence rate ratios (IRR), 95% confidence intervals and *p*-values were calculated. A *p*-value ≤ 0.05 was considered significant. An IRR of <1 indicated a decrease and >1 indicated an increase in the number of antimicrobials to which an isolate was resistant.

## 3. Results

### 3.1. Prevalence of Antimicrobial Resistance

**Figure 1:**
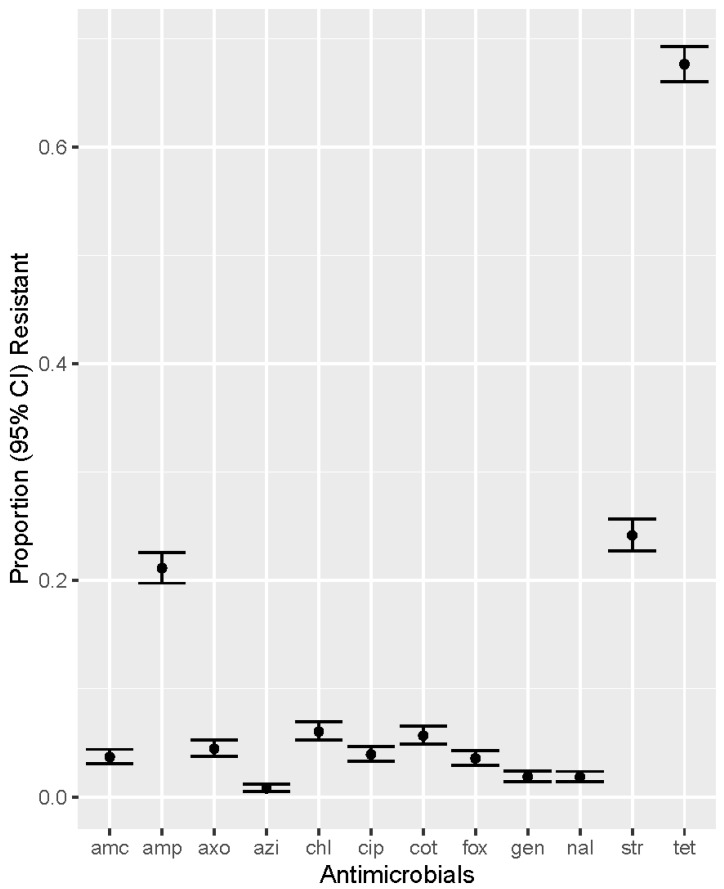
Prevalence of resistance to twelve antimicrobials in commensal *Escherichia coli* (n = 3237) isolated from swine at slaughter across the US, 2013–2019. AMC—Amoxicillin-Clavulanic acid; AMP—Ampicillin; AXO—Ceftriaxone; AZI—Azithromycin; CHL—Chloramphenicol; CIP—Ciprofloxacin; COT—Trimethoprim-Sulfamethoxazole; FOX—Cefoxitin; GEN—Gentamicin; NAL—Nalidixic Acid; STR—Streptomycin; TET—Tetracycline.

Overall, among the 3237 *E. coli* isolates, a very high prevalence of resistance for tetracycline (67.62%), and high resistance for streptomycin (24.13%), and ampicillin (21.10%) were identified (Figure 1). A low level of resistance was observed to nalidixic acid (1.82%), gentamicin (1.85%), cefoxitin (3.55%), amoxicillin-clavulanic acid (3.68%), ciprofloxacin (3.92%), ceftriaxone (4.45%), chloramphenicol (6.02%), and trimethoprim-sulfamethoxazole (5.65%). A very low proportion of isolates (0.77%) showed decreased susceptibility to azithromycin (Figure 1).

### 3.2. Antimicrobial Resistance Trends over Time

**Figure 2:**
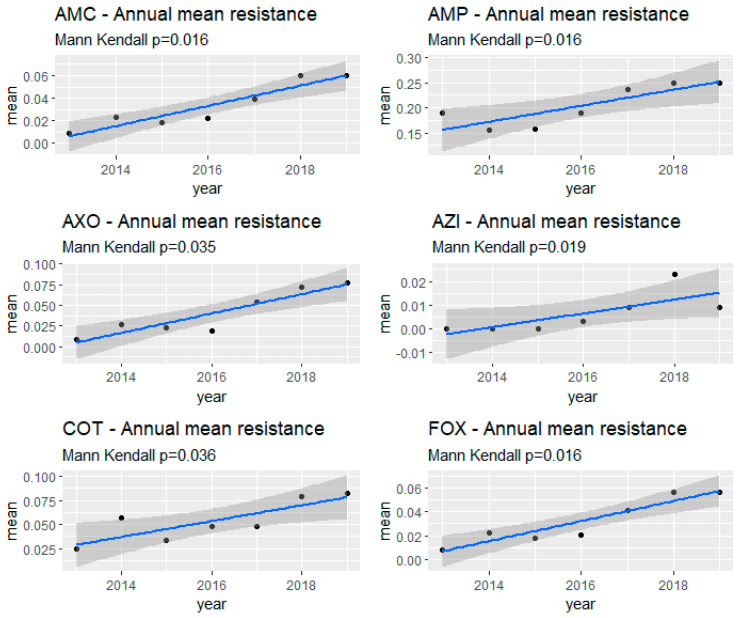
Antimicrobial resistance trends in commensal *Escherichia coli* (n = 3237) isolated from swine at slaughter across the US, 2013–2019. AMC—Amoxicillin-Clavulanic acid; AMP—Ampicillin; AXO—Ceftriaxone; AZI—Azithromycin; COT—Trimethoprim-Sulfamethoxazole; FOX—Cefoxitin.

The Mann–Kendall test (MKT) identified significant monotonous temporal trends over the study period (2013–2019) in the proportion of resistances to amoxicillin-clavulanic acid (*p* = 0.02), ampicillin (*p* = 0.02), ceftriaxone (*p* = 0.04), azithromycin (*p* = 0.02), trimethoprim-sulfamethoxazole (*p* = 0.04), and cefoxitin (*p* = 0.02). For all of these antimicrobials, a linear trendline showed an increasing trend in the proportion of resistance (Figure 2).

### 3.3. Antimicrobial Resistance Patterns and Clustering

#### 3.3.1. Clustering Dendrogram

**Figure 3:**
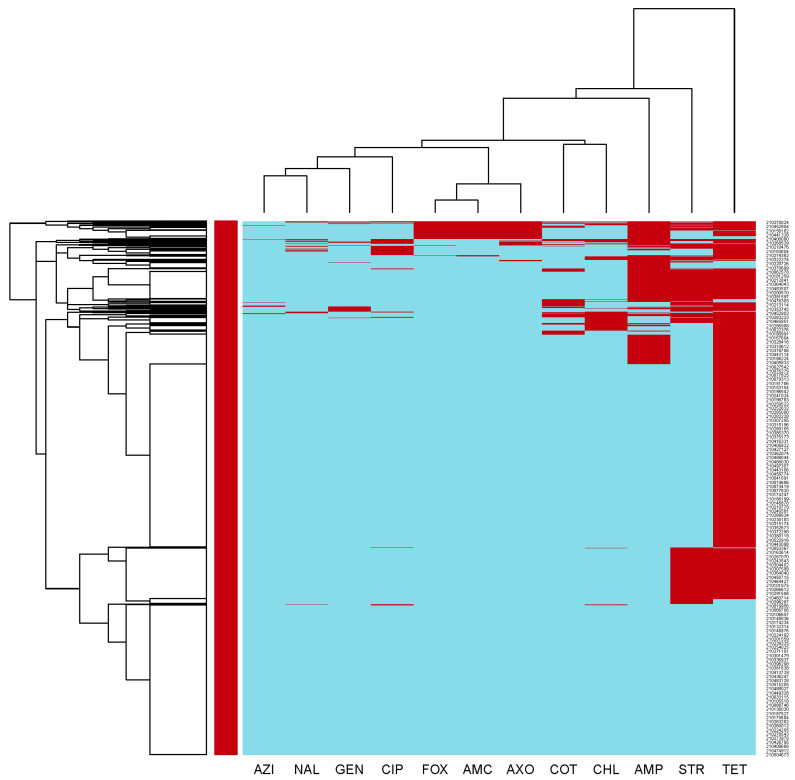
Clustering dendrogram (heatmap) of antimicrobial resistance in commensal *Escherichia coli* isolated from swine at slaughter across the US, 2013–2019. AMC—Amoxicillin-Clavulanic acid; AMP—Ampicillin; AXO—Ceftriaxone; AZI—Azithromycin; CHL—Chloramphenicol; CIP—Ciprofloxacin; COT—Trimethoprim-Sulfamethoxazole; FOX—Cefoxitin; GEN—Gentamicin; NAL—Nalidixic Acid; STR—Streptomycin; TET—Tetracycline.

The clustering dendrogram (heatmap) for resistances to individual antimicrobials in *E. coli* isolates is shown in Figure 3. A cluster of isolates susceptible to all of the tested antimicrobials can be seen at the bottom section of the heatmap rows. A second cluster located above the first cluster includes isolates resistant to streptomycin and tetracycline. In the middle section of the heat map rows, a third cluster includes isolates that were resistant to tetracycline. At the top section of the heatmap rows, several smaller clusters that were resistant to multiple antimicrobials are located.

#### 3.3.2. Correlation among Antimicrobial Resistances

**Figure 4:**
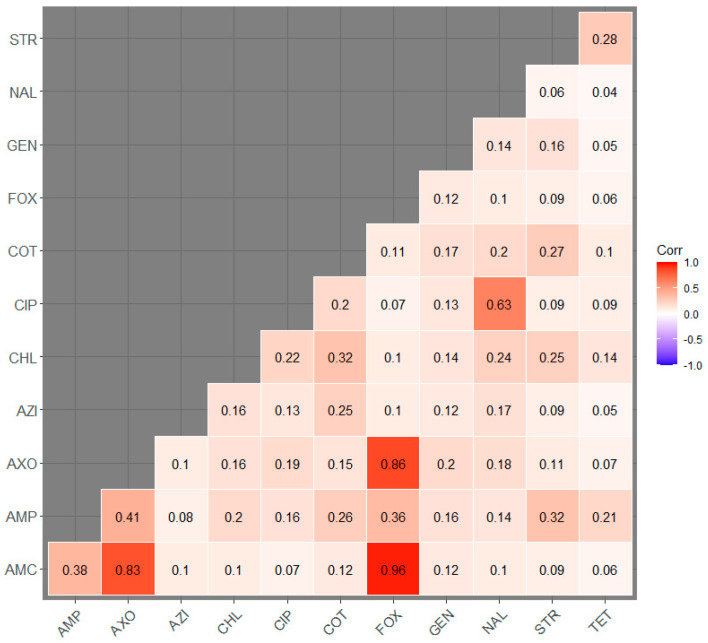
Pearson correlation coefficient heatmap of resistance among twelve antimicrobials in *Escherichia coli* isolates of swine. AMC—Amoxicillin-Clavulanic acid; AMP—Ampicillin; AXO—Ceftriaxone; AZI—Azithromycin; CHL—Chloramphenicol; CIP—Ciprofloxacin; COT—Trimethoprim-sulfamethoxazole; FOX—Cefoxitin; GEN—Gentamicin; NAL—Nalidixic Acid; STR—Streptomycin; TET—Tetracycline.

The Pearson correlation coefficients between resistances to individual antimicrobials are illustrated in Figure 4. The highest correlation coefficients were identified between cefoxitin and amoxicillin-clavulanic acid (0.96), cefoxitin and ceftriaxone (0.86), ceftriaxone and amoxicillin-clavulanic acid (0.83), and nalidixic acid and ciprofloxacin (0.63).

#### 3.3.3. Antimicrobial Resistance Patterns

Table 1 shows the AMR and multidrug resistance (MDR) patterns of *E. coli* isolates. Multidrug resistance was detected in 14.9% (n = 483) (95% CI = 13.7–16.2%) of the *E. coli* isolates from the cecal samples of swine at slaughter. Ninety-eight MDR profiles (resistant to three or more classes of antimicrobials) were identified. According to our findings, the most common MDR pattern was the concurrent resistance to ampicillin-streptomycin-tetracycline (n = 165). In this study, a total of eighteen isolates were found to exhibit resistance to six different antimicrobial classes. Furthermore, four isolates also demonstrated resistance to seven different classes of antimicrobials. (Table 1). It should be noted that of all *E. coli* isolates tested, 27.9% (n = 905) were susceptible to all the tested antimicrobials (Table 1).

The less common AMR and MDR patterns in *E. coli* isolates are described in Table S1.

### 3.4. Poisson Regression Analysis

The results of the Poisson regression model are shown in Table 2. Compared to 2013 the number of antimicrobials to which an *E. coli* isolate was resistant was significantly higher in 2017 (Incidence Rate Ratio (IRR) = 1.24), 2018 (IRR = 1.42), and 2019 (IRR = 1.34).

**Figure 5:**
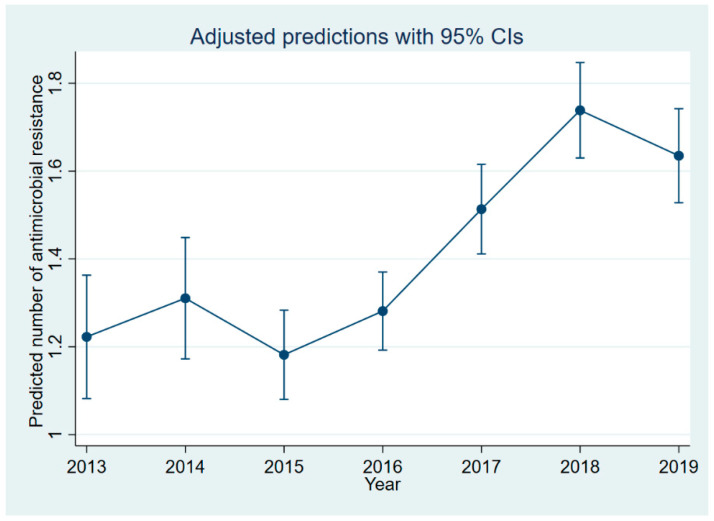
Poisson regression model result of the predicted number of antimicrobials to which an *E. coli* isolate was resistant each year.

The predicted marginal effects for the number of antimicrobials to which an *E. coli* isolate was resistant increased over the study period, showing the highest predictions for 2018, 2019, and 2017 (Figure 5).

## 4. Discussion

Assessing longitudinal surveillance data on AMR in commensal *E. coli* of swine at slaughter is an effective approach to identifying changes in resistance to individual and multiple antimicrobials and detecting emerging trends. Currently, antimicrobial stewardship programs are being implemented in the US swine and other livestock industries to limit the emergence of AMR in commensal and pathogenic enteric bacteria. However, the impact of the implemented interventions and their potential contribution to reducing AMR remains to be assessed. The findings of this study provide evidence-based data on AMR trends in *E. coli* isolated from swine at slaughter, which will aid animal and public health stakeholders in improving antimicrobial stewardship programs.

The current investigation determined that the *E. coli* isolates exhibited very high resistance to tetracycline, and the trend of resistance to tetracycline was not significantly changing over the study period. Comparable resistance profiles have been previously documented in swine-derived *E. coli* isolates from the United States [35] and other countries worldwide [36,37,38,39]. In a prior investigation performed in Belgium [26], a very high level of resistance (>50%) to ampicillin, ciprofloxacin, nalidixic acid, sulfamethoxazole, and tetracycline was reported in commensal *E. coli* strains derived from pigs. In contrast, in the present study, tetracycline was the sole antibiotic demonstrating such a high prevalence of resistance (67.6%), which is an encouraging outcome compared to the aforementioned investigation. Another important finding of this study was the high resistance levels to ampicillin and streptomycin. Similar observations have been reported in long-term antimicrobial resistance monitoring programs conducted in the US [35] and globally [40]. In our study, the prevalence of resistance to streptomycin did not show a temporal trend. On the other hand, a significantly increasing trend in the proportion of resistance to ampicillin was noted, from a low of around 15% in 2014 and 2015 to a high of 25% in 2018 and 2019, which is a concerning finding. In our study only commensal *E. coli* was assessed, however, the transmission of resistance genes between commensal and pathogenic enteric bacteria has been documented before [41]. Therefore, the increase in resistance to ampicillin might impact the treatment efficacy of important swine infectious diseases as ampicillin is used to treat swine respiratory infections and swine colibacillosis [42].

An overall very low level of resistance (4.4%) was observed in our study to extended-spectrum cephalosporins (ceftriaxone), a critically important antimicrobial class for both human and veterinary medicine. However, a concerning increasing temporal trend in resistance to ceftriaxone was identified. While the prevalence of resistance to ceftriaxone was only 0.8% in 2013 and varied between 1.9% and 2.6% between 2014 and 2016, in the later years of our study a higher prevalence was observed (5.4% in 2017, 7.2% in 2018, and 7.7% in 2019). The resistance to extended-spectrum cephalosporins is a multifaceted phenomenon that is primarily mediated by extended-spectrum beta-lactamases (ESBLs). These ESBLs are commonly encoded by the *bla*_TEM_, *bla*_SHV_, and *bla*_CTX-M_ genes. Of these, the *bla*_CTX-M_ genes have been reported as the most prevalent ESBL genes worldwide in both humans and animals [43]. Furthermore, recent reports have indicated the emergence of ESBL genes in bacteria derived from food animals in the US over the past decade [44], which have also been identified in *E. coli* from farm animals across the globe [45,46,47,48].

In our study, a low level of resistance was observed to fluoroquinolones (ciprofloxacin and nalidixic acid), and no temporal trends were observed in their resistance, which is an encouraging finding. The development of resistance to fluoroquinolones is largely attributed to the accumulation of multiple chromosomal mutations in specific genes (*gyrA*, *gyrB*, *parE*, and *parC*). Moreover, certain plasmid-mediated quinolone resistance genes, such as *qnr*, can further contribute to the resistance phenotype. The upregulation of efflux pumps also confers varying resistance levels to this antimicrobial class [49].

Even though we observed a low prevalence of resistance to ceftriaxone a critically important antimicrobial in human medicine the increasing temporal trend in its resistance is more concerning than the high prevalence of resistance to tetracycline and other less important antimicrobials in human medicine [26]. Present literature has consistently demonstrated a positive association between heightened antimicrobial exposure in livestock and an elevated prevalence of resistance in commensal *E. coli* [7,50,51,52,53,54]. Thus, it is imperative to exercise prudence in administering antimicrobial agents, especially in livestock species such as swine. In addition, there is a need to use a One Health approach and identify other risk factors for AMR than AMU, and develop antimicrobial stewardship policies in veterinary medicine to stop the emergence of AMR.

Multidrug resistance was detected in 14.9% of the swine *E. coli* isolates in the present study, which was lower than what was reported for pigs (53.7%) in the previous nationwide surveillance between 1950 and 2002 in the US [35] as well as in the European-wide investigation between 2004 and 2018 (23.7%) [40]. In addition to geographical factors and differences in laboratory and analytical methods used for detecting AMR, the MDR rates and AMR patterns of enteric bacteria in food animals can also be influenced by the antimicrobial use policies and interventions employed in different regions [55]. Various factors, such as the frequency, duration, and dose of antimicrobial use and the types of antimicrobial agents used, can impact the development and spread of AMR [7,56]. Therefore, it is crucial to consider the impact of antimicrobial use policies and interventions when comparing the resistance patterns of enteric bacteria in food animals from different regions.

The present investigation indicated a significant increasing trend in the proportion of resistance of three classes of antimicrobials, including β-lactams, folate inhibitors, and macrolides among commensal *E. coli* isolates from swine at slaughter between 2013 and 2019. A prior US nationwide monitoring investigation has demonstrated an upward trend in the proportion of streptomycin resistance among *E. coli* isolates obtained from food animals over time [35]. The study [35] also reported that *E. coli* isolates from food animals did not exhibit a consistent monotonic resistance trend for ceftriaxone, ciprofloxacin, and nalidixic acid. Our finding agrees with the fluoroquinolone resistance; however, we detected an increasing trend in the proportion of resistance to ceftriaxone. Contrary to these findings, a significant reduction in resistance to ciprofloxacin, nalidixic acid, and tetracycline in commensal *E. coli* isolated from pigs was previously reported in a study conducted in Belgium between 2011 and 2014 [26]. Numerous potential reasons might exist for the increase or decrease of AMR trends in a particular population. The most apparent is the selection pressure on bacteria when exposed to antimicrobial agents. Evidence of significant associations between the utilization of certain antimicrobials and the emergence of antimicrobial resistance in pathogenic and commensal bacteria of swine has already been demonstrated at the farm- level [7,52]. However, due to the ban on using critically important antimicrobials in human medicine in the US food animals since 2017 [57], these increasing resistance trends cannot be explained by the selection pressure of AMU, and other factors might contribute to these phenomena. Co-selection for resistance might play a role when a single drug has the potential to select for resistance to multiple chemically unrelated agents. Additionally, genes responsible for resistance to these compounds are frequently associated with mobile genetic elements, leading to co-resistance [58,59]. Another plausible explanation for the observed trends of increasing resistance in the absence of certain antimicrobial use is that the selection of resistance by a particular compound can potentially result in resistance to other molecules within the same class [40].

Our investigation involved a cluster analysis of AMR in commensal *E. coli* isolates, which revealed diverse clusters of resistances and susceptibility to the antimicrobial agents tested. Notably, certain *E. coli* isolate clusters exhibited resistance to streptomycin and tetracycline-antibiotics that have historically been used in livestock production to treat bacterial infections [60]. These findings underscore the importance of employing a cautious approach toward prescribing commonly used antimicrobials to minimize the development and spread of AMR and MDR among enteric bacteria associated with swine production [7]. Additionally, it is important to implement herd health management and biosecurity practices that decrease the risk of infections and consequently reduce the need of using antimicrobials [61].

The highest correlation coefficients in the present investigation were identified between cefoxitin and amoxicillin-clavulanic acid (0.96), cefoxitin and ceftriaxone (0.86), ceftriaxone and amoxicillin-clavulanic acid (0.83) respectively. The possible reason for such findings could be that all these antibiotics belong to the beta-lactam class. Beta-lactam antibiotics share a similar mechanism of action, which involves inhibiting bacterial cell wall synthesis [62]. Furthermore, high levels of cross-resistance among various beta-lactam antibiotics usually imply that bacteria exhibiting resistance to a particular beta-lactam antibiotic are highly likely to exhibit resistance to other antibiotics within the same class [63]. The co-occurrence of resistance to multiple beta-lactam antibiotics is often due to the presence of plasmids carrying genes that encode multiple types of resistance mechanisms. These plasmids can spread rapidly among bacterial populations, contributing to the widespread resistance to beta-lactam antibiotics [64].

Our findings also indicated that the number of antimicrobials to which an *E. coli* isolate was resistant had increased in 2017, 2018, and 2019 when compared to 2013. The results of our study align with a previous investigation conducted in India, which reported a 1.6-fold increase in MDR *E. coli* isolates derived from food animals between 1980 and 2018 [65]. Contrasting results were also reported in a prior European investigation, wherein a tendency towards a reduction in MDR *E. coli* strains over time was observed in pigs [40]. The variations in antimicrobial use and husbandry practices at the farm level might partly explain differences in AMR between the years in the present investigation. In addition, the higher utilization of crucial antimicrobial agents for therapeutic purposes after their prohibition as growth promoters can be another probable hypothesis. Both hypotheses emphasize the importance of further investigations, especially at the farm level to validate our findings and scrutinize other potentially associated factors in the emergence of AMR and MDR in commensal *E. coli* within the US swine industry.

The present study is not without limitations, including the unequal distribution of isolates across each year and the potential for isolate selection bias. Additionally, the lack of available information on prior antibiotic therapy of swine before slaughter did not allow us to identify factors impacting the prevalence of AMR and the increasing trends of resistance to certain critically important antimicrobials in commensal *E. coli* sourced from swine at slaughter. Finally, data covering a longer period after the implementation of the antimicrobial stewardship measures could assess the effectiveness of these interventions and update our study results.

## 5. Conclusions

This study provides valuable information regarding the prevalence and trends of AMR in commensal *E. coli* isolated from swine at slaughter. Although low levels of resistance to critically important antimicrobials (e.g., third-generation cephalosporins) among commensal *E. coli* isolates was a promising finding, the increasing trend in their resistance despite the existence of AMU reduction interventions is concerning, and warrants further investigation. Such information can support decision-makers in providing guidelines and taking appropriate control measures to reduce AMR in swine production.

## Figures and Tables

**Table 1:** Most common antimicrobial resistance patterns in commensal *E. coli* (n = 3007) isolated from cecal swine samples at slaughter across the United States, 2013–2019.

Antimicrobial Resistance Pattern ^a^	N (%)	Number of Classes	MDR ^b^ (Yes/No)
Susceptible	905 (27.95)	-	-
TET	1109 (34.26)	1	N
STR	30 (0.92)	1	N
AMP	33 (1.02)	1	N
AMP-TET	180 (5.56)	2	N
CHL-TET	27 (0.83)	2	N
COT-TET	19 (0.58)	2	N
CIP-TET	22 (0.68)	2	N
STR-TET	307 (9.48)	2	N
AMP-CHL-TET	9 (0.28)	3	Y
AMP-COT-STR	13 (0.4)	3	Y
AMP-STR-TET	165 (5.09)	3	Y
GEN-STR-TET	9 (0.28)	2	N
COT-STR-TET	17 (0.52)	3	Y
CIP-STR-TET	9 (0.28)	3	Y
CHL-STR-TET	31 (0.95)	3	Y
AMP-CHL-STR-TET	20 (0.62)	4	Y
AMP-COT-STR-TET	16 (0.49)	4	Y
AMC-AMP-AXO-FOX	16 (0.49)	1	N
AMC-AMP-AXO-FOX-TET	34 (1.05)	2	N
AMP-CHL-COT-STR-TET	12 (0.37)	5	Y
AMC-AMP-AXO-FOX-STR-TET	15 (0.46)	3	Y
AMC-AMP-AXO-COT-FOX-STR-TET	9 (0.27)	4	Y

^a^ AMC—Amoxicillin-Clavulanic acid; AMP—Ampicillin; AXO—Ceftriaxone; AZI—Azithromycin; CHL—Chloramphenicol; CIP—Ciprofloxacin; COT—Trimethoprim-Sulfamethoxazole; FOX—Cefoxitin; GEN—Gentamicin; NAL—Nalidixic Acid; STR—Streptomycin; TET—Tetracycline. ^b^ MDR: Multidrug-resistant (Resistant to three or more antimicrobial classes).

**Table 2:** Results of a Poisson regression model showing differences between years in the number of antimicrobials to which an *E. coli* isolate is resistant.

Year	Incidence Rate Ratio	*p*-Value	95% CI ^a^ Low	95% CI High
2013	Referent		-	-
2014	1.07	0.383	0.92	1.25
2015	0.97	0.642	0.84	1.12
2016	1.05	0.494	0.92	1.20
2017	1.24	0.002	1.08	1.41
2018	1.42	<0.001	1.25	1.62
2019	1.34	<0.001	1.17	1.53

^a^ CI: Confidence Interval.

## Data Availability

Data used in this study is publicly available and can be accessed at: https://www.fda.gov/animal-veterinary/national-antimicrobial-resistance-monitoring-system/integrated-reportssummaries (accessed on 1 March 2023).

## Supplementary Materials

The following supporting information can be downloaded at: https://www.mdpi.com/article/10.3390/microorganisms11041033/s1, Table S1: Less common antimicrobial resistance patterns in commensal *Escherichia coli* (n = 230) isolated from cecal swine samples at slaughter across the United States, 2013–2019.

## References

[1] McEwen S.A., Collignon P.J. (2018). Antimicrobial Resistance: A One Health Perspective. Antimicrob. Resist. Bact. Livest. Companion Anim..

[2] Meemken D., Cuny C., Witte W., Eichler U., Staudt R., Blaha T. (2008). Occurrence of MRSA in pigs and in humans involved in pig production—Preliminary results of a study in the northwest of Germany. DTW Dtsch. Tierarztl. Wochenschr..

[3] Chantziaras I., Boyen F., Callens B., Dewulf J. (2014). Correlation between veterinary antimicrobial use and antimicrobial resistance in food-producing animals: A report on seven countries. J. Antimicrob. Chemother..

[4] Centers for Disease Control and Prevention, U.S. Department of Health and Human Services (2019). Antibiotic Resistance Threats in the United States, 2019.

[5] Food and Drug Administration (2018). NARMS 2017 Integrated Report: Executive Summary. U.S. Department of Health and Human Services, Food and Drug Administration. https://www.fda.gov/animal-veterinary/national-antimicrobial-resistance-monitoring-system/2018-narms-update-integrated-report-summary.

[6] Peng Z., Hu Z., Li Z., Zhang X., Jia C., Li T., Dai M., Tan C., Xu Z., Wu B. (2022). Antimicrobial Resistance and Population Genomics of Multidrug-Resistant *Escherichia coli* in Pig Farms in Mainland China. Nat. Commun..

[7] Varga C., Rajić A., McFall M.E., Reid-Smith R.J., Deckert A.E., Checkley S.L., McEwen S.A. (2009). Associations between reported on-farm antimicrobial use practices and observed antimicrobial resistance in generic fecal Escherichia coli isolated from Alberta finishing swine farms. Prev. Vet. Med..

[8] Food and Drug Administration 2021 Summary Report on Antimicrobials Sold or Distributed for Use in Food-Producing Animals. https://www.fda.gov/media/163739/download.

[9] Food and Drug Administration (2012). Guidance for Industry #209: The Judicious Use of Medically Important Antimicrobial Drugs in Food-Producing Animals.

[10] Food and Drug Administration (2015). Veterinary Feed Directive (VFD). https://www.fda.gov/animal-veterinary/development-approval-process/veterinary-feed-directive-vfd.

[11] Skockova A., Kolackova I., Bogdanovicova K., Karpiskova R. (2015). Characteristic and Antimicrobial Resistance in Escherichia coli from Retail Meats Purchased in the Czech Republic. Food Control.

[12] United States Department of Agriculture (USDA) Foreign Agricultural Service. https://apps.fas.usda.gov/psdonline/app/index.html#/app/advQuery.

[13] Gibbons J.F., Boland F., Egan J., Fanning S., Markey B.K., Leonard F.C. (2016). Antimicrobial Resistance of Faecal Escherichia coli Isolates from Pig Farms with Different Durations of In-Feed Antimicrobial Use. Zoonoses Public Health.

[14] Bonardi S., Cabassi C.S., Manfreda G., Parisi A., Fiaccadori E., Sabatino A., Cavirani S., Bacci C., Rega M., Spadini C. (2022). Survey on Carbapenem-Resistant Bacteria in Pigs at Slaughter and Comparison with Human Clinical Isolates in Italy. Antibiotics.

[15] Wasyl D., Hoszowski A., Zając M., Szulowski K. (2013). Antimicrobial Resistance in Commensal *Escherichia coli* Isolated from Animals at Slaughter. Front. Microbiol..

[16] Song H.J., Kim S.J., Moon D.C., Mechesso A.F., Choi J.H., Kang H.Y., Boby N., Yoon S.S., Lim S.K. (2022). Antimicrobial Resistance in Escherichia coli Isolates from Healthy Food Animals in South Korea, 2010–2020. Microorganisms.

[17] Zhang R.Q., Ying G.G., Su H.C., Zhou L.J., Liu Y.S. (2013). Antibiotic Resistance and Genetic Diversity of Escherichia coli Isolates from Traditional and Integrated Aquaculture in South China. J. Environ. Sci. Health B.

[18] Khan F.M., Gupta R. *Escherichia coli* (*E. coli*) as an Indicator of Fecal Contamination in Groundwater: A Review. Proceedings of the ICSDWE2020.

[19] Alvarez-Fernandez E., Cancelo A., Diaz-Vega C., Capita R., Alonso-Calleja C. (2013). Antimicrobial resistance in *E. coli* isolates from conventionally and organically reared poultry: A comparison of agar disc diffusion and sensi test gram-negative methods. Food Control.

[20] Burow E., Rostalski A., Harlizius J., Gangl A., Simoneit C., Grobbel M., Kollas C., Tenhagen B.A., Käsbohrer A. (2019). Antibiotic Resistance in *Escherichia coli* from Pigs from Birth to Slaughter and Its Association with Antibiotic Treatment. Prev. Vet. Med..

[21] Stubberfield E., AbuOun M., Sayers E., O’Connor H.M., Card R.M., Anjum M.F. (2019). Use of whole genome sequencing of commensal *Escherichia coli* in pigs for antimicrobial resistance surveillance, United Kingdom, 2018. Eurosurveillance.

[22] McEwen S.A., Fedorka-Cray P.J. (2002). Antimicrobial Use and Resistance in Animals. Clin. Infect. Dis..

[23] Anjum M.F., Schmitt H., Börjesson S., Berendonk T.U., WAWES Network (2021). The Potential of Using *E. coli* as an Indicator for the Surveillance of Antimicrobial Resistance (AMR) in the Environment. Curr. Opin. Microbiol..

[24] Diallo O.O., Baron S.A., Abat C., Colson P., Chaudet H., Rolain J.M. (2020). Antibiotic Resistance Surveillance Systems: A Review. J. Glob. Antimicrob. Resist..

[25] Hesp A., Veldman K., van der Goot J., Mevius D., van Schaik G. (2019). Monitoring Antimicrobial Resistance Trends in Commensal *Escherichia coli* from Livestock, the Netherlands, 1998 to 2016. Eurosurveillance.

[26] Hanon J.B., Jaspers S., Butaye P., Wattiau P., Méroc E., Aerts M., Imberechts H., Vermeersch K., Van der Stede Y. (2015). A Trend Analysis of Antimicrobial Resistance in Commensal *Escherichia coli* from Several Livestock Species in Belgium (2011–2014). Prev. Vet. Med..

[27] Mader R., Muñoz Madero C., Aasmäe B., Bourély C., Broens E.M., Busani L., Callens B., Collineau L., Crespo-Robledo P., Damborg P. (2022). Review and Analysis of National Monitoring Systems for Antimicrobial Resistance in Animal Bacterial Pathogens in Europe: A Basis for the Development of the European Antimicrobial Resistance Surveillance Network in Veterinary Medicine (EARS-Vet). Front. Microbiol..

[28] Food and Drug Administration (2021). NARMS Now.

[29] DANMAP (2020). Use of Antimicrobial Agents and Occurrence of Antimicrobial Resistance in Bacteria from Food Animals, Food and Humans in Denmark. https://www.danmap.org/Reports/2020.aspx.

[30] Government of Canada (2018). Canadian Integrated Program for Antimicrobial Resistance Surveillance (CIPARS) 2018 Annual Report. https://www.canada.ca/en/public-health/services/publications/drugs-health-products/canadian-integrated-program-antimicrobial-resistance-surveillance-2018-annual-report.html.

[31] Hayer S.S., Rovira A., Olsen K., Johnson T.J., Vannucci F., Rendahl A., Perez A., Alvarez J. (2020). Prevalence and trend analysis of antimicrobial resistance in clinical *Escherichia coli* isolates collected from diseased pigs in the USA between 2006 and 2016. Transbound. Emerg. Dis..

[32] Food and Drug Administration (2020). The National Antimicrobial Resistance Monitoring System Manual of Laboratory Methods.

[33] European Food Safety Authority (2021). The European Union Summary Report on Antimicrobial Resistance in zoonotic and indicator bacteria from humans, animals and food in 2018/2019. EFSA J..

[34] R Core Team (2020). R: A Language and Environment for Statistical Computing.

[35] Tadesse D.A., Zhao S., Tong E., Ayers S., Singh A., Bartholomew M.J., McDermott P.F. (2012). Antimicrobial Drug Resistance in *Escherichia coli* from Humans and Food Animals, United States, 1950–2002. Emerg. Infect. Dis..

[36] Gong J., Xu M., Zhu C., Miao J., Liu X., Xu B., Zhang J., Yu Y., Jia X. (2013). Antimicrobial resistance, presence of integrons and biofilm formation of *Salmonella* Pullorum isolates from Eastern China (1962–2010). Avian Pathol..

[37] Chen X., Zhang W., Yin J., Zhang N., Geng S., Zhou X., Wang Y., Gao S., Jiao X. (2014). *Escherichia coli* isolates from sick chickens in China: Changes in antimicrobial resistance between 1993 and 2013. Vet. J..

[38] Yassin A.K., Gong J., Kelly P., Lu G., Guardabassi L., Wei L., Han X., Qiu H., Price S., Cheng D. (2017). Antimicrobial resistance in clinical *Escherichia coli* isolates from poultry and livestock, China. PLoS ONE.

[39] Zhang P., Shen Z., Zhang C., Song L., Wang B., Shang J., Yue X., Qu Z., Li X., Wu L. (2017). Surveillance of antimicrobial resistance among *Escherichia coli* from chicken and swine, China, 2008–2015. Vet. Microbiol..

[40] De Jong A., El Garch F., Hocquet D., Prenger-Berninghoff E., Dewulf J., Migura-Garcia L., Perrin-Guyomard A., Veldman K.T., Janosi S., Skarzynska M. (2022). European-Wide Antimicrobial Resistance Monitoring in Commensal *Escherichia coli* Isolated from Healthy Food Animals between 2004 and 2018. J. Antimicrob. Chemother..

[41] Sunde M., Sørum H. (2001). Self-Transmissible Multidrug Resistance Plasmids in *Escherichia coli* of the Normal Intestinal Flora of Healthy Swine. Microb. Drug Resist..

[42] Misumi W., Funamori T., Hamada K., Iwamoto J., Fujisono S., Chitose K., Kusumoto M. (2021). Association between Antimicrobial Treatment and Resistance of Pathogenic *Escherichia coli* Isolated from Diseased Swine in Kagoshima Prefecture, Japan. J. Vet. Med. Sci..

[43] Bevan E.R., Jones A.M., Hawkey P.M. (2017). Global Epidemiology of CTX-M β-Lactamases: Temporal and Geographical Shifts in Genotype. J. Antimicrob. Chemother..

[44] Wittum T.E., Mollenkopf D.F., Erdman M.M. (2012). Detection of *Salmonella* enterica Isolates Producing CTX-M Cephalosporinase in U.S. Livestock Populations. Appl. Environ. Microbiol..

[45] Guo Y.-F., Zhang W.-H., Ren S.-Q., Yang L., Lü D.-H., Zeng Z.-L., Liu Y.-H., Jiang H.-X. (2014). IncA/C Plasmid-Mediated Spread of CMY-2 in Multidrugresistant *Escherichia coli* from Food Animals in China. PLoS ONE.

[46] Ferreira J.C., Penha Filho R.A.C., Andrade L.N., Berchieri Junior A., Darini A.L.C. (2017). Diversity of Plasmids Harboring blaCMY-2 in Multidrug-Resistant *Escherichia coli* Isolated from Poultry in Brazil. Diagn. Microbiol. Infect. Dis..

[47] Castellanos L.R., Donado-Godoy P., León M., Clavijo V., Arevalo A., Bernal J.F., Timmerman A.J., Mevius D.J., Wagenaar J.A., Hordijk J. (2017). High Heterogeneity of *Escherichia coli* Sequence Types Harbouring ESBL/ AmpC Genes on IncI1 Plasmids in the Colombian Poultry Chain. PLoS ONE.

[48] Castellanos L.R., van der Graaf-van Bloois L., Donado-Godoy P., Mevius D.J., Wagenaar J.A., Hordijk J., Zomer A.L. (2019). Phylogenomic Investigation of IncI1-I Plasmids Harboring blaCMY-2 and blaSHV-12 in *Salmonella* enterica and *Escherichia coli* in Multiple Countries. Antimicrob. Agents Chemother..

[49] Holmes A.H., Moore L.S.P., Sundsfjord A., Steinbakk M., Regmi S., Karkey A., Guerin P.J., Piddock L.J.V. (2016). Understanding the Mechanisms and Drivers of Antimicrobial Resistance. Lancet.

[50] Berge A.C., Atwill E.R., Sischo W.M. (2005). Animal and Farm Influences on the Dynamics of Antibiotic Resistance in Faecal *E. coli* in Young Dairy Calves. Prev. Vet. Med..

[51] Bosman A.B., Wagenaar J.A., Stegeman J.A., Vernooij J.C., Mevius D.J. (2014). Antimicrobial Resistance in Commensal *E. coli* in Veal Calves Is Associated with Antimicrobial Drug Use. Epidemiol. Infect..

[52] Burow E., Simoneit C., Tenhagen B.A., Käsbohrer A. (2014). Oral Antimicrobials Increase Antimicrobial Resistance in Porcine *E. coli*—A Systematic Review. Prev. Vet. Med..

[53] Li P., Wu D., Liu K., Suolang S., He T., Liu X., Wu C., Wang Y., Lin D. (2014). Investigation of antimicrobial resistance in *E. coli* and Enterococci isolated from Tibetan pigs. PLoS ONE.

[54] Simoneit C., Burow E., Tenhagen B.A., Käsbohrer A. (2015). Oral administration ofantimicrobials increase antimicrobial resistance in *E. coli* from chicken-asystematic review. Prev. Vet. Med..

[55] Callens B., Cargnel M., Sarrazin S., Dewulf J., Hoet B., Vermeersch K., Wattiau P., Welby S. (2018). Associations between a Decreased Veterinary Antimicrobial Use and Resistance in Commensal *Escherichia coli* from Belgian Livestock Species (2011–2015). Prev. Vet. Med..

[56] Shrestha R.D., Agunos A., Gow S.P., Deckert A.E., Varga C. (2022). Associations between Antimicrobial Resistance in Fecal *Escherichia coli* Isolates and Antimicrobial Use in Canadian Turkey Flocks. Front. Microbiol..

[57] Gens K.D., Singer R.S., Dilworth T.J., Heil E.L., Beaudoin A.L. (2022). Antimicrobials in Animal Agriculture in the United States: A Multidisciplinary Overview of Regulation and Utilization to Foster Collaboration: On Behalf of the Society of Infectious Diseases Pharmacists. Open Forum Infect. Dis..

[58] Sharma P., Haycocks J.R.J., Middlemiss A.D., Kettles R.A., Sellars L.E., Ricci V., Piddock L.J.V. (2017). The multiple antibiotic resistance operon of enteric bacteria controls DNA repair and outer membrane integrity. Nat. Commun..

[59] Partridge S.R., Kwong S.M., Firth N., Jensen S.O. (2018). Mobile Genetic Elements Associated with Antimicrobial Resistance. Clin. Microbiol. Rev..

[60] WOAH (World Organisation for Animal Health) (2021). OIE Annual Report on Antimicrobial Agents Intended for Use in Animals. https://www.oie.int/en/what-we-do/monitoring/antimicrobial-resistance/annual-report-on-antimicrobial-agents/.

[61] Postma M., Vanderhaeghen W., Sarrazin S., Maes D., Dewulf J. (2017). Reducing Antimicrobial Usage in Pig Production without Jeopardizing Production Parameters. Zoonoses Public Health.

[62] Bush K., Jacoby G.A., Medeiros A.A. (1995). A functional classification scheme for beta-lactamases and its correlation with molecular structure. Antimicrob. Agents Chemother..

[63] Livermore D.M. (1995). Beta-lactamases in laboratory and clinical resistance. Clin. Microbiol. Rev..

[64] de Oliveira D.V., Nunes L.S., Barth A.L., Van Der Sand S.T. (2017). Genetic background of β-lactamases in Enterobacteriaceae isolates from environmental samples. Microb. Ecol..

[65] Pires J., Huisman J.S., Bonhoeffer S., Van Boeckel T.P. (2022). Increase in Antimicrobial Resistance in *Escherichia coli* in Food Animals between 1980 and 2018 Assessed Using Genomes from Public Databases. J. Antimicrob. Chemother..

