# Supplementary material for: Evaluating Antimicrobial Resistance Trends in Commensal Escherichia coli Isolated from Cecal Samples of Swine at Slaughter in the United States, 2013–2019
Source: Microorganisms. 2023 Apr 15;11(4):1033. doi: 10.3390/microorganisms11041033 (PMC10142105; doi:10.3390/microorganisms11041033)
Supplement: Supplementary file 1 [file microorganisms-11-01033-s001.zip › microorganisms-2334137-supplementary.pdf]

Supplementary Table S1. Less common antimicrobial resistance patterns in commensal *Escherichia coli* (n = 230) isolated from cecal swine samples at slaughter across the United States, 2013-2019.

Antimicrobial resistance pattern [a]	n=230 (%)	Number of classes	MDR(Y/N)
GEN	1 (0.03)	1	N
COT	1 (0.03)	1	N
CIP	3 (0.09)	1	N
CHL	2 (0.06)	1	N
AMP-COT	1 (0.03)	2	N
AMP-STR	6 (0.18)	2	N
CHL-STR	3 (0.09)	2	N
CHL-CIP	1 (0.03)	2	N
CIP-NAL	2 (0.06)	1	N
COT-STR	2 (0.06)	2	N
CIP-STR	1 (0.03)	2	N
AZI-TET	3 (0.09)	2	N
AZI-STR	1 (0.03)	2	N
NAL-TET	1 (0.03)	2	N
GEN-TET	1 (0.03)	2	N
GEN-STR	2 (0.06)	1	N
GEN-AMP	3 (0.09)	2	N
AMP-AXO	2 (0.06)	1	N
AMC-AMP-TET	4 (0.12)	2	N
AMP-AXO-STR	1 (0.03)	2	N
AMP-AXO-TET	3 (0.09)	2	N
AMP-CHL-COT	1 (0.03)	3	Y
AMP-CHL-STR	2 (0.06)	3	Y
AMP-CIP-NAL	2 (0.06)	2	N
AMP-CIP-TET	6 (0.18)	3	Y
AMP-COT-TET	5 (0.15)	3	Y
AMP-GEN-TET	1 (0.03)	3	Y
AMP-GEN-STR	1 (0.03)	2	N
FOX-STR-TET	1 (0.03)	3	Y
AZI-COT-TET	1 (0.03)	3	Y
CIP-NAL-TET	7 (0.21)	2	N
CIP-NAL-STR	2 (0.06)	2	N
CHL-COT-TET	5 (0.15)	3	Y
CHL-CIP-TET	1 (0.03)	3	Y
CHL-CIP-NAL	1 (0.03)	3	Y
AMC-AMP-COT-STR	1 (0.03)	3	Y
AMC-AMP-FOX-TET	2 (0.06)	2	N
AMC-AMP-STR-TET	1 (0.03)	3	Y
AMP-AXO-CIP-NAL	1 (0.03)	2	N

AMP-AXO-STR-TET	1 (0.03)	3	Y
AMP-AZI-COT-TET	1 (0.03)	4	Y
AMP-CHL-COT-STR	2 (0.06)	4	Y
AMP-CHL-COT-TET	5 (0.15)	4	Y
AMP-CIP-NAL-TET	3 (0.09)	3	Y
AMP-CIP-STR-TET	5 (0.15)	4	Y
AMP-COT-GEN-TET	1 (0.03)	4	Y
AMP-GEN-STR-TET	3 (0.09)	3	Y
AMP-CIP-COT-TET	2 (0.06)	4	Y
CIP-NAL-STR-TET	1 (0.03)	3	Y
COT-GEN-STR-TET	2 (0.06)	3	Y
CHL-NAL-STR-TET	1 (0.03)	4	Y
CHL-GEN-STR-TET	2 (0.06)	3	Y
CHL-COT-STR-TET	5 (0.15)	4	Y
CHL-COT-NAL-TET	2 (0.06)	4	Y
CHL-CIP-STR-TET	1 (0.03)	4	Y
AZI-COT-STR-TET	2 (0.06)	4	Y
AXO-FOX-STR-TET	1 (0.03)	3	Y
AMC-AMP-AXO-FOX-STR	2 (0.06)	2	N
AMC-AMP-AXO-FOX-GEN	1 (0.03)	2	N
AMC-AMP-FOX-STR-TET	1 (0.03)	3	Y
AMP-AXO-CHL-STR-TET	1 (0.03)	4	Y
AMP-AXO-CIP-GEN-TET	4 (0.12)	4	Y
AMP-AXO-COT-STR-TET	5 (0.15)	4	Y
AMP-AXO-GEN-STR-TET	1 (0.03)	3	Y
AMP-CHL-GEN-STR-TET	3 (0.09)	4	Y
AMP-CIP-COT-GEN-NAL	1 (0.03)	4	Y
AMP-CHL-CIP-NAL-TET	1 (0.03)	4	Y
AMP-CIP-NAL-STR-TET	2 (0.06)	4	Y
AMP-CIP-COT-STR-TET	3 (0.09)	5	Y
CHL-CIP-NAL-STR-TET	2 (0.06)	4	Y
CHL-CIP-COT-STR-TET	3 (0.09)	5	Y
CHL-CIP-COT-NAL-TET	1 (0.03)	4	Y
AMP-COT-GEN-STR-TET	5 (0.15)	4	Y
AZI-CHL-COT-STR-TET	1 (0.03)	5	Y
AZI-COT-GEN-STR-TET	1 (0.03)	4	Y
CHL-CIP-COT-NAL-TET-STR	1 (0.03)	5	Y
AZI-CHL-CIP-COT-NAL-TET	1 (0.03)	5	Y
AMC-AMP-AXO-CHL-FOX-TET	2 (0.06)	3	Y
AMC-AMP-AXO-CIP-FOX-TET	1 (0.03)	3	Y
AMC-AMP-AXO-COT-FOX-TET	2 (0.06)	3	Y
AMC-AMP-AXO-CIP-FOX-NAL	1 (0.03)	2	N
AMP-CIP-COT-NAL-STR-TET	2 (0.06)	5	Y
AMP-AXO-CIP-GEN-NAL-TET	1 (0.03)	4	Y

AMP-CHL-COT-GEN-STR-TET	1 (0.03)	5	Y
AMP-CHL-CIP-COT-STR-TET	4 (0.12)	6	Y
AMP-CHL-CIP-COT-NAL-TET	1 (0.03)	5	Y
AMP-AXO-CIP-NAL-STR-TET	1 (0.03)	4	Y
AMP-AXO-CHL-GEN-STR-TET	1 (0.03)	4	Y
AMP-AXO-CHL-COT-STR-TET	1 (0.03)	5	Y
AMP-AXO-CHL-CIP-STR-TET	2 (0.06)	5	Y
AMP-AXO-CHL-CIP-NAL-TET	1 (0.03)	4	Y
AMP-AXO-CHL-CIP-COT-TET	1 (0.03)	5	Y
AMP-AZI-COT-GEN-STR-TET	1 (0.03)	5	Y
AMP-AZI-CHL-COT-STR-TET	3 (0.09)	6	Y
AMC-AMP-AXO-CHL-FOX-STR-TET	6 (0.18)	4	Y
AMC-AMP-AXO-FOX-GEN-STR-TET	1 (0.03)	3	Y
AMC-AMP-AXO-CIP-FOX-NAL TET	3 (0.09)	3	Y
AMC-AMP-AXO-AZI-FOX-STR-TET	2 (0.06)	4	Y
AMP-CHL-CIP-COT-NAL-STR-TET	3 (0.09)	6	Y
AMP-AXO-CHL-CIP-NAL-STR-TET	1 (0.03)	5	Y
AMP-AXO-CHL-CIP-GEN-NAL-TET	2 (0.06)	5	Y
AMP-AXO-AZI-CHL-CIP-COT-TET	1 (0.03)	6	Y
AMP-AZI-CHL-CIP-COT-NAL-STR-TET	2 (0.06)	7	Y
AMP-AXO-CHL-CIP-COT-NAL-STR-TET	2 (0.06)	6	Y
AMC-AMP-AXO-COT-FOX-GEN-STR-TET	1 (0.03)	4	Y
AMC-AMP-AXO-CHL-FOX-GEN-STR-TET	2 (0.06)	4	Y
AMC-AMP-AXO-CHL-COT-FOX-GEN-STR	1 (0.03)	4	Y
AMC-AMP-AXO-CHL-CIP-FOX-STR-TET	1 (0.03)	5	Y
AMC-AMP-AXO-AZI-CHL-FOX-STR-TET	1 (0.03)	5	Y
AMC-AMP-AXO-CHL-COT-FOX-GEN-STR-TET	2 (0.06)	5	Y
AMC-AMP-AXO-CHL-CIP-COT-FOX-STR-TET	1 (0.03)	6	Y
AMC-AMP-AXO-CHL-CIP-COT-FOX-NAL-TET	1 (0.03)	5	Y
AMP-AZI-CHL-CIP-COT-GEN-NAL-STR-TET	1 (0.03)	7	Y
AMC-AMP-AXO-AZI-CHL-CIP-COT-FOX-NAL-TET	1 (0.03)	6	Y
AMC-AMP-AXO-CHL-CIP-COT-FOX-GEN-NAL-STR-TET	2 (0.06)	6	Y
AMC-AMP-AXO-AZI-CIP-COT-FOX-GEN-NAL-STR-TET	1 (0.03)	6	Y
AMC-AMP-AXO-AZI-CHL-CIP-COT-FOX-GEN-NAL-STR-TET	1 (0.03)	7	Y

[a] AMC- Amoxicillin-Clavulanic acid; AMP- Ampicillin; AXO-Ceftriaxone; AZI- Azithromycin; CHL-Chloramphenicol; CIP- Ciprofloxacin; COT- Trimethoprim-Sulfamethoxazole; FOX- Cefox-itin; GEN-Gentamicin; NAL- Nalidixic Acid; STR- Streptomycin; TET- Tetracycline.
